# 

**Published:** 1889-09

**Authors:** 


### Society Meetings.

THE AMERICAN PEDIATRIC SOCIETY will hold its annual meeting in Washington, D. C., September 20 and 21, 1889.

---

THE AMERICAN GYNECOLOGICAL SOCIETY will hold its annual meeting in Boston, September 17, 18, and 19, 1889.

---

THE MISSISSIPPI VALLEY MEDICAL SOCIETY will hold its annual meeting at Evansville, Ind., September 10, 11, and 12, 1889.

---

THE ASSOCIATION OF AMERICAN PHYSICIANS will hold its annual meeting at Washington, D. C., September 18, 19, and 20, 1889.

THE AMERICAN SOCIETY OF MICROSCOPISTS held a very successful meeting in this city, August 20–23, 1889. Dr. George E. Fell, of Buffalo, was elected president for the ensuing year.

---

THE AMERICAN ASSOCIATION OF OBSTETRICIANS AND GYNECOLOGISTS will hold its annual meeting at the Burnet House, Cincinnati, September 17, 18, and 19, 1889. The programme is an exceedingly interesting one, and all interested are invited to attend.

### BOOKS AND PAMPHLETS RECEIVED.

Transactions American Surgical Association for 1889.

Essentials of Pathology and Morbid Anatomy. By C. E. Armand Semple. With forty-six illustrations. Philadelphia: W. B. Saunders, 1889.

On Disordered Digestion and Dyspepsia. Frank Woodbury, A. M., M. D. George S. Davis, Detroit, Mich., 1889.

Prolapse of the Womb, with Especial Reference to the [so-called] Hypertrophic Elongation of the Supra-Vaginal Portion of the Cervix, with report of a case. By Lewis H. Adler, Jr., M. D. Reprint from the *Medical News*, August 3, 1889.

Electrical Distribution of Heat, Light and Power. By Harold P. Brown, Electrical Engineer. With partial list of deaths from electrical lighting apparatus, and an address by John Murray Mitchell, Counselor-at-Law on Legislative Control of Dangerous Electrical Currents. New York: Press of J. W. Pratt & Son, 1889.

Cornell University, College of Agriculture. Bulletin of the Agricultural Experiment Station, Horticultural Department. VII. July, 1889. On the Influences of Certain Conditions upon the Sprouting of Seeds.

Paring and Suture Clamp Forceps. By Edmond V. Joye, M. D., Atlanta, Ga. Reprint from the New York *Medical Journal* for July 13, 1889.

Value and Proper Use of Arsenic in Skin Diseases. By George H. Rohé, M. D., Baltimore, Md. Reprint from the Virginia *Medical Monthly*, January, 1888.

The Electrolytic Decomposition of Organic Tissues. By George H. Rohé, M. D. Reprint from the New York *Medical Journal* for December 1, 1888.

On the Diagnosis of Pregnancy in the Early Months. By Llewellyn Eliot, M. D., Washington, D. C. Reprint from the *Journal of the American Medical Association*, June 22, 1889. Chicago.

Expression in the Treatment of Trachoma. By A. E. Prince, M. D., Jacksonville, Ill. Reprint.

Pelvic and Abdominal Drainage. By David Prince, M. D., Jacksonville, Ill. Reprint.

Four cases of Gunshot Wound of the Abdomen Treated by Laparatomy. With remarks. By David Barrow, M. D., Lexington, Ky. Reprint from the *Journal of the American Medical Association*, June 15, 1889. Chicago.

President's Message and Recommendation, and annual address before the Texas State Medical Association at San Antonio, April, 1889, by J. F. Y. Paine, M. D., Galveston, Texas. Reprint from Transactions T. S. M. A.

Twenty-second Annual Report of the Health Department of the Board of Public Affairs to the Honorable Common Council of the City of Cincinnati, for the year ending December 31, 1881. Byron Stanton, M. D., Health Officer. Cincinnati, 1889.

Admission of Utah. Report of the Committee on Territories on the Admission of Utah as a State, to the House of Representatives, second session, Fiftieth Congress. Washington: Government Printing Office, 1889.

Report for the Year 1888–89, presented by the Board of Managers of the Observatory of Yale University to the President and Fellows.

Merck's Bulletin, June, 1889, July, 1889.

The Annual Report of the Department for the Insane of the Pennsylvania Hospital. For the year ending fourth month, 22d, 1889. Philadelphia: Press of Ferris Bros.

Annual Report of Morse Dispensary of Cooper Medical College for 1888. San Francisco: W. A. Woodard & Co., Printers, 1889.

Report of resident physician of Brigham Hall, a hospital for the insane, for the year 1888. Canandaigua, N. Y., 1889.

Annual announcement of the Buffalo College of Pharmacy, Department of Pharmacy, University of Buffalo. For the session of 1889–90.

Boston University School of Medicine, seventeenth annual announcement and catalogue. July, 1889.

The Central University of Kentucky. Medical Department. Hospital College of Medicine, Louisville, Kentucky. Circular for session 1890.

The Central University of Kentucky. Dental Department. Louisville College of Dentistry, session IV., 1890.

Announcement Gross Medical College, of Denver. Medical Department of the Rocky Mountain University. Session 1889–90.

Cooper Medical College, San Francisco. Annual announcement. Session of 1889.

Seventeenth annual announcement of the Physio-Medical College of Indiana. Indianapolis. Session of 1889–90.

College of Physicians and Surgeons in the City of New York. Medical Department of Columbia College. Annual catalogue and announcement. New York, June, 1889.

The Medico-Chirurgical College of Philadelphia. Announcement for sessions of 1889–90.

Thirteenth annual announcement and catalogue of the Hahnemann Medical College and Hospital of Chicago, Ill., 1889–90.

State Board of Health Bulletin, Nashville, Tenn., July 15, 1889.

Abstract of Proceedings of the Michigan State Board of Health, April, 1889.

Monthly Bulletin of the New York State Board of Health, June, 1889.

Health in Michigan, July, 1889.

Report of Deaths and Contagious Diseases for the month of July, 1889. Newport, R. I.

Weekly Abstract of Sanitary Reports. Vol. IV. Nos. 31, 32, 33, 34.

---

MESSRS. JOHN WYETH & BRO.'S advertisement, in this issue, is worthy of the careful attention of our patrons. They give a complete list of their Compressed Hypodermic Tablets—embracing in all some seventy-one different agents and combinations—the most complete we have yet seen. In it will be found almost every medicament used in hypodermic practice. This house was the first to devise this most valuable and convenient form of subcutaneous medication. Its well-known reputation is a sufficient guarantee for all the claims they make for these as well as for all their preparations so widely and favorably known.

---

BROMIDIA.—I have used the Bromidia (Battle), and the results obtained have been really excellent. It certainly combines all the advantages of other preparations of this nature, while, at the same time, it possesses none of their disadvantages. The fact that it produces no unpleasant sensation on awaking renders it specially valuable.

DR. LUD. MARC.

ST. NAZAIRE-SUR-LOIRE, FRANCE.

### Literary and Other Notes.

THE *Boston Advertiser* issued August 29th, Dr. Oliver Wendell Holmes' birthday, a special paper, containing a special article on the "Autocrat" by Frank B. Sanford, his personal friend, letters from all his surviving college classmates, Harvard, 1829, and other features which make it the feature of Boston journalism this Summer.

---

F. A. DAVIS, of Philadelphia, has in press a new work on the "Practical Applications of Electricity in Medicine and Surgery, by Dr. C. A. Liebig, Jr., of Johns Hopkins University, and Prof. George H. Rohé, of the College of Physicians and Surgeons, of Baltimore.

The part on Physical Electricity, written by Dr. Liebig, one of the recognized authorities on the science in the United States, will treat fully such topics of interest as Storage Batteries, Dynamos, the Electric Light and the Principles and Practice of Electrical Measurement in their Relations to Medical Practice.

Prof. Rohé, who writes on Electro-Therapeutics, discusses at length the recent developments of electricity in the treatment of stricture, enlarged prostate, uterine fibroids, pelvic cellulitis, and other diseases of the male and female genito-urinary organs.

The applications of electricity in dermatology, as well as in the diseases of the nervous system, are also fully considered.

The work will be fully illustrated by engravings and original diagrams.

---

THERE is no other exhibit of the class in the United States section to rival that of William R. Warner & Co. at the Paris Exposition. From the globe-advertising merchant comes an exhibit which the native pharmaciens can look at with both admiration and wonderment. The display is enough to make any Frenchman curious, and their arrangement such as to be above deprecatory criticism; and those Frenchmen there could not be a people with better taste for the proper and harmonious exhibition of products. A glance through their own magnificent section of pharmacy will verify this. Readers would find superfluous a description in detail of the Messrs. Warner's essentially fine installation covering all their soluble sugar-coated pills, salts, &c. Suffice it to remark that at the Paris Universelle their exhibit is thoroughly representative, comprises all the makers' fabrications, and is decidedly an honor to the concern.—*Pharmaceutical Record.*

### UNIVERSITY OF THE STATE OF NEW YORK.

### EXAMINATIONS ON SUBJECTS PREPARATORY TO THE STUDY OF MEDICINE, AS REQUIRED BY CHAPTER 468, LAWS OF 1889.

1. The Legislature, during its recent session, enacted the following law to elevate the standard for admission to practice medicine:

AN ACT to provide for the preliminary education of medical students.

APPROVED by the Governor June 13, 1889. PASSED, three-fifths being present.

*The People of the State of New York, represented in Senate and Assembly, do enact as follows:*

SECTION 1. Before the Regents of the University of the State of New York or the trustees of any medical school or college within this State shall confer the degree of doctor of medicine on any person, who has not received a baccalaureate degree in course from a college or university duly authorized to confer the same, they shall require him to file with the secretary or recording officer of their university or college a certificate showing that, prior to entering upon the prescribed three years' study of medicine, he passed an examination conducted under the authority and in accordance with the rules of the Regents of the University of the State of New York, in arithmetic grammar, geography, orthography, American history, English composition, and the elements of natural philosophy, and such certificate shall be signed by the secretary of the regents and countersigned by the principal or commissioner conducting said examination.

SEC. 2. This act shall not apply to persons who have already entered upon the prescribed three years' study of medicine, nor shall it alter the time of study or the courses of medical instruction required to be pursued in the medical colleges of this State by existing statutes.

SEC 3. This act shall take effect immediately.

2. For the accommodation of candidates under the above law, special examinations will be held at the times and places noted in the annexed schedule. All correspondence on this subject should be addressed to Edward I. Devlin, A. M., the Commissioner in charge of such examinations, at the Regents' office, Albany, N. Y.

3. All candidates must notify the commissioner, by letter, at least one week in advance, stating at what place and in what studies they wish to be examined. No fee will be charged, and candidates will be informed of the result within twenty days from the close of the examination.

4. To insure success, the candidate should have a thorough knowledge of the whole of a standard school text book on each of the required subjects, but cube root will not be included in the arithmetic examination.

5. Printed question papers will be issued from the office of the Regents for each examination. The answers must be written in ink on legal cap paper, and arranged and numbered in the same order as the questions. Candidates should bring paper, pen, ink, and blotter.

6. Seventy-five per cent. of correct answers is required in all subjects except orthography. In the latter study, the candidate must spell correctly eighty-five out of one hundred words, such as are commonly used in correct literature.

7. In the examination of the arithmetic, the entire operation required for obtaining the result must be given, and the answer or result must be reduced to the simplest form.

8. The candidate must write at the top of the first page of his answer-paper (1) the place of examination, (2) the subject, (3) the date, (4) his own full name, (5) his post-office address.

9. At the conclusion of his answers on any subject, or at the expiration of the time allotted for such subject, the candidate must make and subscribe to the declaration given below, by writing on the lines immediately succeeding the last answer the words: *I do so declare*, and then his name. Every paper lacking this declaration and signature will be rejected. The declaration to which he thus subscribes is as follows:

*Form of Declaration.*

☞ Do you now, at the close of the examination in arithmetic (etc., as the case may be), conscientiously declare that you had no knowledge of the questions to be proposed, that you have neither given to any other candidate, nor received from any source, explanations or other aid in answering any of them, and that you have not spent more than the allowed time. If so, write *in the next line after the end of your set of answers*, near the right side of the paper, the words,

*"I do so declare."*

and underneath, *subscribe your name.*

10. All papers which fall below the required standard will be returned to the candidate. For those accepted, pass cards, certifying such proficiency, will be issued, and when all the subjects are completed, the certificate provided for in article 14 will be sent.

11. Candidates may offer at any trial one or more of the subjects required, and the subjects passed at such trial will be placed to their credit on the records of the Regents. In like manner, subjects passed in the regular Regents' examinations in the academies will be allowed and credited to candidates. Should a candidate allow an interval of five years to elapse without passing an additional subject, he will be deemed to have relinquished his candidacy, and will be dropped from the records.

12. Examinations in the subjects required by the above law, also form a part of the system established by the Regents and conducted in the 308 academical institutions under their visitation throughont the State. The dates for the current academic year are November 18–22, 1889; January 20–24, 1890; March 3–7, 1890; June 9–13, 1890.

13. While candidates will find the special examinations better adapted to their purpose, they may enter the examinations held in the academies; *provided*, in all cases, that they make application to the principal at least two weeks in advance, and pass the examinations in the several subjects at the same time and under the same regulations, as the candidates in attendance at such academies; and *provided*, that they pay to such academies a fee of one dollar for each subject entered for examination. The answer–papers of persons so examined will be reviewed at the Regents' office in the same order as those of pupils in regular attendance, but they will lose no time by necessary delay at this office, if their papers are satisfactory.

14. Whenever all the subjects required have been passed by a candidate, and he has mailed to the commissioner, at Albany, a claim specifying when and where each subject was passed, the Regents will grant him a special certificate, known as the *Medical Student's Certificate.* This certificate is to be signed by the commissioner or principal conducting the examination. It will not be issued to pupils in regular atrendance at an academy, but only to those who have finished their academic course and are actually entering upon the study of medicine.

15. The above instructions are subject to such changes as may from time to time be deemed necessary.

EDWARD I. DEVLIN,

*Commissioner.*

MELVIL DEWEY,

*Secretary.*

AUGUST 20, 1889.

---

SCHEDULE OF EXAMINATIONS, 1889-'90.

[Extract ]

Buffalo, September 30–October 2, 1889, February 4–6, 1890, at the Buffalo High School.

All candidates for these special examinations must notify Commissioner Devlin at Albany, by letter, at least one week in advance, stating at what place and in what studies they wish to be examined.

